# How is aid used to exert power? Gender equality promotion and migration control in Senegal

**DOI:** 10.1002/jid.3650

**Published:** 2022-03-25

**Authors:** Iliana Olivié

**Affiliations:** ^1^ Department of Applied & Structural Economics & History Complutense University of Madrid Spain; ^2^ Elcano Royal Institute Madrid Spain

**Keywords:** aid, development cooperation, EU, gender equity, influence, migration, power, Senegal

## Abstract

This article describes how aid‐influence mechanisms previously identified by academic literature (aid conditionality, tied aid, consultants, people‐to‐people exchanges and the support of like‐minded donors) are triggered in a selection of six aid projects implemented by Spain and Germany and involving the EU in Senegal, in the domains of gender equity and migration control. Aid‐influence nexuses might prove ineffective if there is a lack of political will on the part of the partner, an insufficient involvement of its Administration or local actors, a mis‐selection of people involved in the aid‐influence link, or if the scale of the project is too small.

## 1 INTRODUCTION

Academic literature on development cooperation tends to assume that international aid involves a power relation, where the donor country has the ability to exert some type of influence on the partner or recipient. 1 However, not many studies have addressed the precise mechanisms of this aid‐power nexus, resulting in a short list of factors applicable to bilateral aid spaces: aid conditionality, tied aid, consultants, people‐to‐people exchanges and the support of like‐minded donors (Anderson, 2018; Banerjee & Rondinelli, 2003; Dauvergne & Farias, 2012; De Bruyn, 2016; Deych, 2015; Fejerskov, 2017; Gu et al., 2016; Harmer et al., 2013; Jung et al., 2018; Kwon et al., 2015; Montinola, 2010; Tarte, 2008).

This article explores the aid‐influence mechanisms at work (previously identified by academic literature) in a series of projects implemented by EU donors (the EU, Spain and Germany) in Senegal, in the fields of gender equality promotion 2 and migration control. This work contributes to previous studies by gathering in a single work the (otherwise scattered) whole range of mechanisms in a bilateral aid space and to studies on EU as a global development player by exploring the mechanics of influence through EU aid.

Our study aims to unravel the aid‐influence nexus, showing that this link is not mechanic. On the one hand, for aid to be a tool for the exertion of international influence, aid projects need to include specific features that might act as transmission belts of interests or values (aid conditionality, tied aid, consultants, people‐to‐people exchanges and the support of like‐minded donors). On the other hand, even with the presence of such features, aid might not be able to facilitate the influence on the part of the donor due to a series of reasons, including the lack of political will on the part of the partner, the insufficient involvement of its Administration or local actors, the mis‐selection of people involved in the aid‐influence link, or the scale of the project.

Section 2 reviews the academic literature on aid and power. Section 3 describes the methodology for filtering six development cooperation projects implemented by Spanish and German cooperation agencies, with the involvement of the EU, in the two fields of gender equity and migration in Senegal. Section 4 presents the results and Section 5 concludes.

## 2 AID AND POWER: INPUTS FROM THE ACADEMIC LITERATURE

According to Gallarotti (2015), ‘the study of international power is still quite underdeveloped relative to its importance in international politics’ (Gallarotti, 2015, p. 247). There are, of course, several definitions of power in international relations, such as Dahl's (1957); ‘A has power over B to the extent that [they] can get B to do something that B would not otherwise do’ (Dahl, 1957, pp. 202–203). However, most studies on power deal with it as part of a country's status in the world scene and not so much in terms of its role or specific actions. Moreover, despite contemporary studies of power differing from their initial Realist root, many still consider power, as the Realist school did, in terms of its sources, assets or resources (for instance, having/not having weapons of mass destruction). The latter applies even to middle powers (de Sá Guimaraes & de Almeida, 2018; Forsberg, 2011) despite their role having been explored through literature on entrepreneurial power (Karim, 2018; Öniş & Kutlay, 2016).

This vacuum has been identified by several authors, who highlight the importance of conceptualising power as a specific relationship that causes behavioural change, rather than as a status or attribute (Covarrubias & Schiavon, 2018; Lagan, 2018; Lim & Ferguson, 2018).

Those that address the issue tend to converge in a similar shortlist of mechanisms for power exertion: economic and military instruments, coercion, attraction or persuasion (propaganda, community framing), diplomacy (strategic coalition building, circumventing formal channels), creating or invoking norms or agendas, 3 leading through example (contagion, emulation or imitation), and bargaining (de Sá Guimaraes & de Almeida, 2018; Forsberg, 2011; Hagström, 2005; Lim & Ferguson, 2018; Riddervold & Rosén, 2015).

These somehow very general mechanisms are similar to those identified by academic literature on aid as a tool for power exertion from a neo‐colonialist lens. As shown by Lagan (2018), neo‐colonialism envisages aid as a zero‐sum gain between donors and recipients, focuses on results or objectives to be shaped (not so much on the mechanisms to do so) and narrows down the analysis to the business front (leaving aside other potential interests or values such as migration control or gender equality).

We therefore now focus on several studies that dig into the specifics of aid and power by identifying a series of concrete mechanisms in the aid universe through which aid is used as a tool for exerting international influence.

These mechanisms might gain strength with aid dependency, since, for instance, according to Banerjee and Rondinelli (2003), the thickness of the aid‐privatisation nexus might be determined by official development assistance (ODA) as a proportion of national income or production (GDP).

Gu et al. (2016) analyse the aid‐influence nexus (the development encounters) between China and Africa by means of six case studies on Chinese companies involved in the agricultural sector in Mozambique and Zimbabwe. In these cases, the aid‐influence glue in the development encounter is made up of what could be described as tied aid in OECD jargon.

As mentioned above, one main field of reflection is the capacity of aid to import–export ideas, paradigms or agendas on development. Take, for instance, the well‐known case of the Washington Consensus and the reforms undertaken in Latin America (and in other developing regions) during the 80s and 90s, in the framework of debt agreements with multilateral organisations such as the World Bank and the International Monetary Fund (IMF) (Williams, 2020). 4 Such agreements often included clauses on privatisation, a feature pointed out by Montinola (2010) and also by Banerjee and Rondinelli (2003) in their studies on the influence of aid on privatisation processes in 35 developing countries in Africa, Asia and Latin America. They concluded that foreign aid has played a major role in privatisation processes only when combined with a strong institutional development in the developing country.

As for the EU, it could be labelled a normative power, a feature consistent with the exertion of influence via aid, and with the aim of defining political and development agendas—one of the main goals identified by the literature on aid and power. This attribute has been extensively studied by academic literature. The Union, as a melting pot of political actors at the national and subnational levels of up to 27 member states (MS), struggles with the difficulties of making varied interests and values converge in a coherent external action where trade, investments or migration targets align with human rights, environmental sustainability or labour standards (Carbone, 2008; Casas‐Cortes et al., 2016; Faber & Orbie, 2009; Orbie & Del Biondo, 2015). 5

In these cases, an important tool for the exertion of power through aid is technical assistance. The importance of this people‐to‐people type of aid is also mentioned by Deych (2015) when exploring the involvement of BRICS countries (Brazil, Russia, India, China and South Africa) in Africa: ‘[…] Hu Jintao declared that China would shift development assistance for Africa from “hard” infrastructure assistance to “soft” assistance, and would stress education, people‐to‐people exchange and joint research’ (Zhang, 2013 quoted in Deych, 2015); as well as by Kwon et al. (2015) when analysing the involvement of the Asian Development Bank (ADB), the IMF and the World Bank in Cambodia; and by Anderson (2018) when studying Africa's health diplomacy.

Several studies on the EU have also underlined this mechanism for the particular case of aid projects in migratory contexts, through international exchanges with policing counterparts (Sandor, 2016), 6 summits, conferences and workshops on migration (Casas‐Cortes et al., 2016; Vives, 2017), knowledge and technology transfers in the field of security (Frowd, 2018).

**FIGURE 1:**
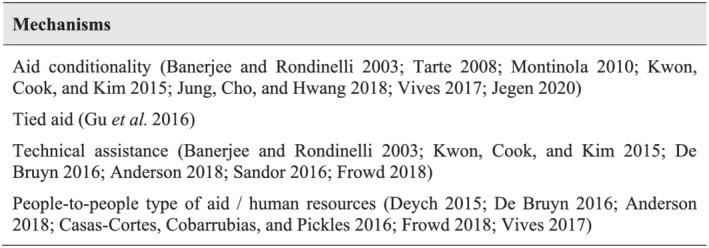
Mechanisms for power or influence through aid: a literature review. *Source*: the author

Aid conditionality has been identified, too, as a major tool of power exertion through aid for exporting political, social and economic paradigms to third countries. But such mechanism needs not be used exclusively with an ideational dimension. Its use can be of a transactional nature, to obtain a political or economic advantage in a different field, as identified by Tarte (2008), who analyses the environmental conditions involved in Japanese aid to developing countries with fishing interests for the donor country. According to Jung et al. (2018), this type of aid conditionality would be similar to that imposed by South Korea when it uses ODA as a mechanism for aligning votes in multilateral organisations. Here too, several works mention EU aid initiatives (including aid forgiveness) subordinated to migration control (Jegen, 2020; Vives, 2017) (Figure 1).

It could be argued that technical assistance is a general area of influence—a sort of ‘space’ for influence—not a specific mechanism in the aid domain. Actually, when pointing out technical assistance as a mechanism of influence, academic literature is referring to the same idea as with the people‐to‐people type of aid, which involves human resources. Technical assistance *per se* does not necessarily leverage influence. It does if it involves exchanges of officials between the two countries, when consultants related to the donor country are appointed in public institutions in the partner country and/or when international aid supports ‘like‐minded’ people or institutions in the partner country with the capacity to lobby for a given political objective or paradigm (for instance, gender equality). In short, previous literature allows us to identify five main mechanisms that donors might have used for shaping ideas or paradigms or for moulding political agendas: (a) aid conditionality, (b) tied aid, (c) consultants, (d) people‐to‐people exchanges and (e) supporting like‐minded actors.

## 3 DEFINING THE CASE STUDY AND ITS METHODOLOGY: GERMANY AND SPAIN IN SEARCH FOR GENDER EQUALITY AND MIGRATION CONTROL IN SENEGAL

Empirically exploring aid as a channel for exerting influence or power on partner countries on the part of donors requires identifying a ‘development encounter’, in Gu et al.'s (2016) words.

In this paper, we aim to explore the EU as a donor. The EU is a highly significant donor, providing approximately half of total ODA, a leading flagship of the Union's external action (EC, 2016). For these reasons, there is a strong case for exploring the EU's capacity for exerting power through aid.

The selection of the partner country in this development encounter should also feature a series of characteristics such as high aid dependency (measured with ODA as a proportion of GDP). Secondly, the donor country (or the group of donors) must have a specific interest besides poverty reduction or human and sustainable development. Such an interest can be linked to development issues in very general terms (e.g., good governance and gender equality agendas) or to other public policies (e.g., commercial interests and migration control). Thirdly, as pointed out in Section 2, aid is only one of several channels through which countries can exert power over others. In order to correctly explore the capacity of aid to serve as a channel for influence, it must be ensured that a large proportion of the donor(s)–partner country relation is limited to aid matters.

Senegal complies with all the above criteria. As for aid dependency, according to OECD‐DAC figures, ODA to the country stood at 6.3% of its GNI in 2019. This ratio is well above the average for all developing countries (0.6% in 2019) and for the Sub‐Saharan region (3% that same year).

Moreover, a great deal of the aid volume comes from the EU. According to the same source, over the 2017–19 period, the EU (including both EU institutions and MSs) provided more than 35% of net ODA in Senegal. EU aid is made up of that of the EU institutions plus the development assistance of individual MS channelled through bilateral and other multilateral channels. Significant EU donors in Senegal include France, EU institutions, Italy, Germany, Luxembourg and Spain. Given that analysing the whole range of EU donors goes well beyond the scope of this paper, it will focus on EU institutions, Germany and Spain.

All three are key stakeholders in the Senegalese development scene. EU institutions have a central role in channelling the EU's political vision regarding Africa. Germany is becoming a development superpower, with increasing aid flows and a strengthened presence in Africa. Spain, a modest and relatively young donor in the EU space is, however, a key player in the tricky intersection between migration and development (given its geographical position) and a vocal country in the gender equality agenda.

### 3.1 Donors' goals

**FIGURE 2:**
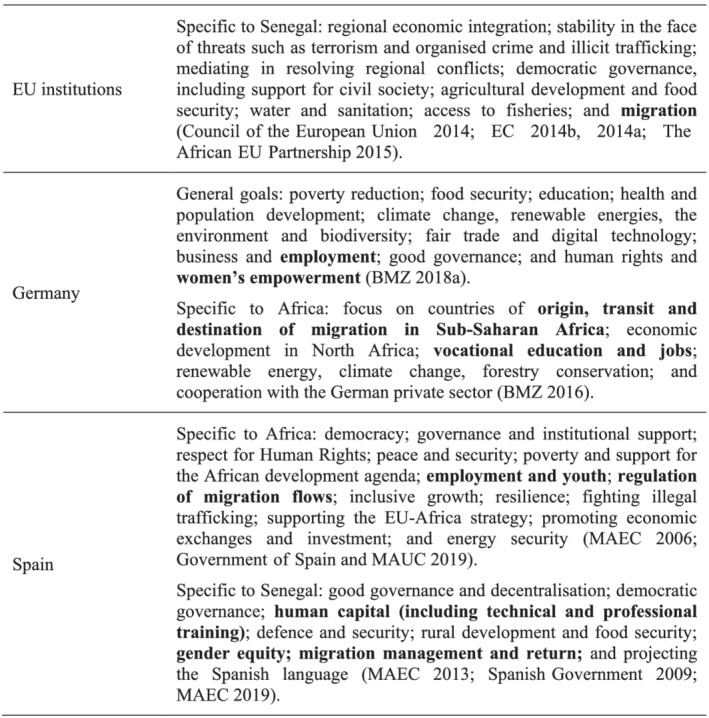
The EU's political and development goals in its relations with Senegal. *Source*: the author

This leads to the second selection criterion for the partner country, which is that donors must have specific interests in the recipient country that go beyond strictly developmental issues such as poverty reduction or human development. Stated interests and goals can be identified in the strategic official documents in force in the period during which this research was conducted (between 2019 and 2021) (see Figure 2).

Besides human development goals such as poverty eradication, in their strategic documents, EU donors emphasise other political goals, such as democratic governance, gender equality, defence and security and migration control.

As for the EU institutions, reflecting the ongoing concerns regarding irregular migration, EU‐Senegal cooperation efforts have continued to focus on measures to counter the issue. The Valletta Summit on Migration (The African EU Partnership, 2015) saw European and African heads of state brought together through the Africa‐EU Partnership with the aim of improving cooperation in the area of migration. It led to a political declaration and the creation of an action plan, which takes in the following broad goals: (a) addressing the root causes of irregular migration and forced displacement; (b) enhancing cooperation on legal migration and mobility; (c) reinforcing the protection of migrants and asylum seekers; (d) preventing and combating irregular migration, migrant smuggling and trafficking in human beings; and (e) working more closely to improve cooperation on return, readmission and reintegration.

Described as the German Federal Ministry for Economic and Development Cooperation's (BMZ – after its name in German) priority continent, in 2014, Germany was actively engaged in 32 African countries, devoting €1200 million annually to development programmes, which accounted for 50% of the country's total bilateral ODA funding.

Education has been singled out as a particular focus for Germany's global development cooperation plans. The Development Policy 2030 document underlines plans to allocate 25% of its ODA funding to education and vocational training (BMZ, 2018a). Two initiatives are highlighted: the Skills Initiative for Africa, run in conjunction with the African Union, and the Special Initiative on Training and Job Creation. The focus on training and employment has a direct link with the migration issue: The BMZ programme ‘Successful in Senegal’ is aimed at the development of skills and is hoped to reduce migration and provide returnees with vocational training and opportunities. In 2018, a German centre for jobs, migration and reintegration was opened in Senegal, partly with the goal of providing information on regular migration options and irregular migration risks. The same year, an additional €5.6 million in funding was announced for projects aimed at returnees (BMZ, 2018b).

Women's rights are one of the 10 priorities defined by Germany in order to comply with the 2030 Agenda: ‘One special focus is women's rights. Across the world, women are taking on responsibility but are still, in many cases, discriminated against and denied their rights’ (BMZ, 2018a, p. 21). When it comes specifically to BMZ's work in Africa, support for skill improvement is conducted with a gender approach, and Germany explicitly welcomes gender equity initiatives undertaken by other EU partners (BMZ, 2016).

Gender equity and migration are the two dimensions selected for Spain's regional cooperation in West Africa, via programmes with the New Partnership for Africa's Development (NEPAD) and the Economic Community of West African States (ECOWAS), respectively. Although gender equity is not one of the main targets of Spain's 2013–17 strategy with Senegal, the document acknowledges the need for a greater emphasis in this dimension, its transversal nature and the need to address it from a perspective of rights, as well as a main part of Spanish cooperation in the area of democratic quality. Conversely, migration management is a top goal according to the same document. It is approached from the security perspective, for instance, via the support for and the training of the security forces for controlling migration inflows and outflows, with Senegal being a major migration hub in the region (MAEC, 2013).

Consistently with the previous strategy, the 2019–23 strategic document for Spanish cooperation with Senegal raises the profile of the gender equality target and maintains migration issues among its top priorities. It becomes a transversal goal for all activities: support for productive activities, access to basic services and the efficiency of public services. Migration is still approached from a security perspective, although, as with other donors, it is now also linked to employment issues (MAEC, 2019).

In short, EU institutions, Germany and Spain claim various development and political objectives, beyond poverty eradication, in their aid relations with Senegal. Several of those political and developmental aims are shared by them; and that is the case with migration management and gender equity. It could be argued that gender equity is a value the EU aims at promoting as a normative power, while migration control would fall in the domain of its domestic interests. Given that influencing through aid might operate differently given this distinction, these two political domains are therefore selected for this case study on the exertion of influence via aid.

### 3.2 Methodology

This paper's aim is to understand the mechanisms through which the EU institutions, Germany and Spain might have used development assistance for exerting influence on Senegal's stakeholders with the aim of introducing and reinforcing the gender equality agenda and/or to gain control over migration flows.

Following the theoretical framework described earlier, the mechanisms for exerting influence through aid can be (a) aid conditionality, (b) tied aid, (c) consultants, (d) people‐to‐people exchanges and/or (e) supporting like‐minded actors.

Empirically observing the occurrence of such mechanisms requires analysing aid projects that might be linked to these two political objectives. Aid conditionality, tied aid at a project level, face‐to‐face interactions such as the inclusion of consultants or official exchanges and support for like‐minded actors can be seen in development projects' descriptions. Moreover, stakeholders from donors and from the partner country involved in such projects can provide valuable information on the capacity of aid projects to trigger political dialogue and, therefore, on influence mechanisms at work (De Bruyn, 2016). Therefore, research techniques for this case study include a combination of the analysis of official documents of aid projects with semi‐structured interviews with key stakeholders, thus also allowing for triangulating information.

Three rounds of over 20 semi‐structured interviews were conducted in Madrid and Dakar between July 2019 and December 2021, including fieldwork in Senegal in November 2019. Meetings were held with local NGOs, the Senegalese authorities, private institutions, multilateral organisations and EU donors. Conversations covered the following issues: (a) the participation of the donor community in Senegal's public policies; (b) the extent to which aid activities involve official exchanges, consultants appointed, the political conditionality of aid and/or the support for local like‐minded actors; (c) the ranking of donors according to their significance; and (d) the potential of aid projects to effectively improve political dialogue between donors and the partner country.

### 3.3 Selected projects

**FIGURE 3:**
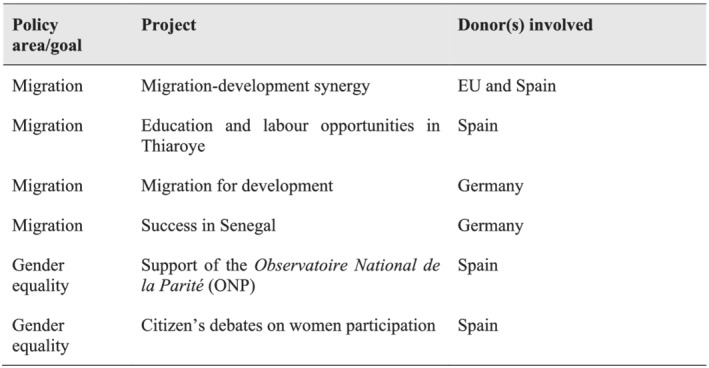
Selected aid projects involving a donor's influence in migration and gender issues. *Source*: the author

In addition to providing valuable information, knowledge and assessments, these interviews also contributed to a more precise definition of the case under study. Given Senegal's high aid dependency ratio, its condition as a regional migration hub and the cultural and sociological elements that facilitate working with a gender equity approach, the portfolio of projects of these European donors with direct connections to migration and gender issues is wide and varied. Since exploring each of these projects greatly exceeds the scope of this paper, the interviews also allowed for the filtering of the main projects where influence mechanisms through aid might have been at work (see Figure 3).

## 4 RESULTS: AID‐INFLUENCE MECHANISMS AT WORK

Of all the projects or programmes underlined by the informants, the EU Emergency Trust Fund for stability and addressing root causes of irregular migration and displaced persons in Africa (EUTF for Africa) is particularly significant. Launched by the EU institutions, it gathers several EU donors active in Senegal (including EU institutions, France and Spain), therefore guaranteeing a set of common migration objectives for the stakeholders.

The EUTF for Africa was one of the tangible results of the Valletta Summit on Migration described earlier. The fund was established to provide additional funding for the implementation of the Valletta action plan and is currently worth over €5 000 million (EC, 2020b).

All in all, the aid budget now committed by the EU in Senegal in the framework of the EUTF for Africa amounts to nearly €171 million distributed in 10 different projects. Over 50% of these funds target greater economic and employment opportunities; the other goals are improved migration management, better governance and conflict prevention and the strengthening of resilience (Altai Consulting, 2020; EC, 2020a).

Spanish cooperation is involved in various activities in the framework of the EUTF in Senegal, one of them being the project for the ‘Support of inclusive governance of migration in Senegal for the improvement of the synergy between migration and development’ 7 (hereafter migration‐development synergy). More precisely, the project is part of a larger one on ‘Strengthening management and governance of migration and return and for sustainable reintegration in Senegal and support for the investments of the Senegalese diaspora’, 8 of almost €28 million, with the participation of Spain, France and the International Organisation for Migration (IOM). There is, therefore, a convergence of political objectives and interests on the part of EU donors and a key member of the multilateral community around migration control.

More precisely, this €9.5 million project on the migration‐development synergy aims at strengthening national strategies on migration policy and the support system for migrants in order to enhance their participation in Senegal's economic and social development.

The project is implemented by the Spanish Agency for International Development Cooperation (*Agencia Española de Cooperación Internacional para el Desarrollo*, AECID) and the (now called) Directorate General for the Support of the Senegalese Overseas (*Direction Générale d'Appui aux Sénégalais de l'Extérieur*, DGASE). That is, Spanish cooperation works in this domain with a like‐minded actor that also happens to be part of the Administration of the partner country. The aim of the cooperation between the two institutions is to strengthen the DGASE's capacities for the territorial definition of Senegal's migration policy and the coherence of other DGASE actions such as the activities conducted with the diaspora inside and outside Senegal. More specifically, there are three targets, the first being to support the final phase of preparation as well as the political approval and initial phases of implementation of Senegal's migration policy. The second target is to strengthen the technical, organisational and material capacities of the state structures responsible for migration management. In this case, it also includes a decentralisation approach as it requires the support for two types of local institutions: the Offices for Reception and Orientation (*Bureaux d'Acceuil et d'Orientation*, BAOS) and the Regional Agencies for Development (*Agences Régionales de Développement*, ARD). BAOS are partly the result of previous support by international cooperation agencies, dating back to the migratory crises of the 2000s. Thirdly, the project supports migrants' organisations and associations in their contribution to integration of migration issues at the local level (AECID, 2017).

**FIGURE 4:**
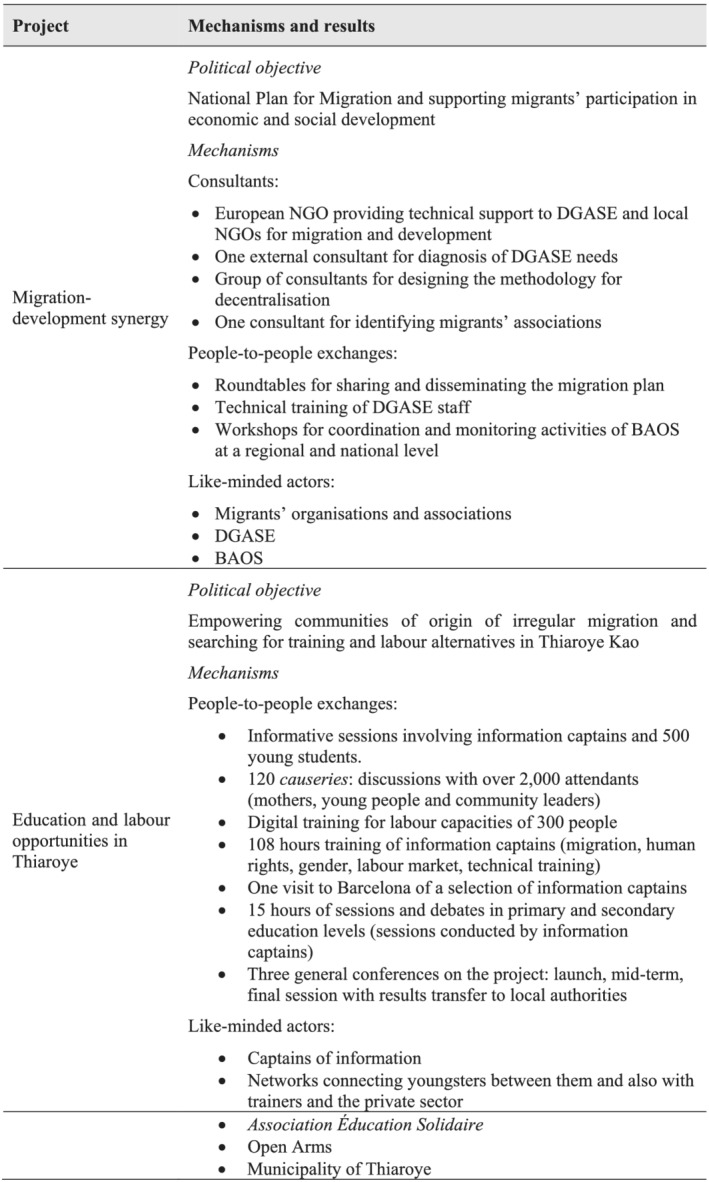
Aid‐influence mechanisms and expected political results in Spanish cooperation migration projects. *Source*: the author

Besides like‐minded actors, which would be the strongest aid‐influence mechanism at work, the project involves other aid‐influence mechanisms such as appointed consultants, roundtables and workshops (see Figure 4). To some extent, it could be argued that some type of tied aid is involved, since one of the institutions providing technical support is a European NGO. In short, all aid‐influence mechanisms identified by academic literature are involved with the exception of aid conditionality.

Spanish cooperation is also implementing development cooperation projects related to migration, besides the activities conducted in the framework of the trust fund. Such is the case with ‘Origin Thiaroye: empowering communities of origin of irregular migration and searching for training and labour alternatives in Thiaroye Kao (Senegal)’ 9 (AECID, 2020b). This is a more limited project, both financially (the budget is under €124 000) and geographically, since it is aimed exclusively at Thiaroye Kao in the Pikine department, an important hub of migration flows. The political objective is similar to that of the project on migration‐development synergy described above. With this activity, AECID aims to reduce the migration outflow, empowering potential migrants by offering local alternatives to migration.

This goal should be achieved through two main lines of action. First, a team of 50 ‘information captains’ are to be trained. Once trained, these like‐minded actors are expected to guide potential migrants in the deconstruction of the migration narrative to Europe and also to explore local alternatives to irregular migration. Secondly, the project is to support the training of 240 youngsters through networks that should enhance the connection between young people, training organisations and the private sector. The capacities of local organisations and governance bodies will also be strengthened.

Here, too, like‐minded actors are a key element of the project, both the captains of information and the networks aimed at connecting human capital, training and economic opportunities, public stakeholders (the municipality of Thiaroye), local associations (*Association Éducation Solidaire*) and the entity Open Arms, which has a strong symbolic role in the project's framework. People‐to‐people exchanges are an important mechanism at work. In addition, the approach is based on the interaction of two mechanisms, like‐minded actors and different forms of people‐to‐people exchanges, since captains of information are expected to interact with trainers, young students, mothers and community leaders (see Figure 4). Consultants are expected to have a limited role (even non‐existent) and, as with the previous project, there is no aid conditionality at work.

Just like Spain and other EU actors, Germany is currently implementing a series of development cooperation projects in Senegal in the field of the migration‐development nexus. The programme on Migration for Development (see Figure 3) was commissioned by the German Federal Ministry for Economic and Development Cooperation (BMZ) to the German Development Agency (GIZ) and is framed under the BMZ project on Returning to New Opportunities. According to donor sources, it started in 2017 and was implemented in 13 countries, including Senegal. The approach of Migration for Development is to improve the social and economic prospects for the Senegalese in order to favour return, in addition to tackling the causes of irregular migration; that is, a very similar approach to the Spanish cooperation project mentioned above. The programme cooperates with local authorities and supports services provided by charities and social agencies that offer training and advice for returnees (GIZ, 2020b).

Under this umbrella, several training and information activities are conducted both in Germany (via charities, municipalities, local associations …) and in migrants' countries of origin (with local training centres).

**FIGURE 5:**
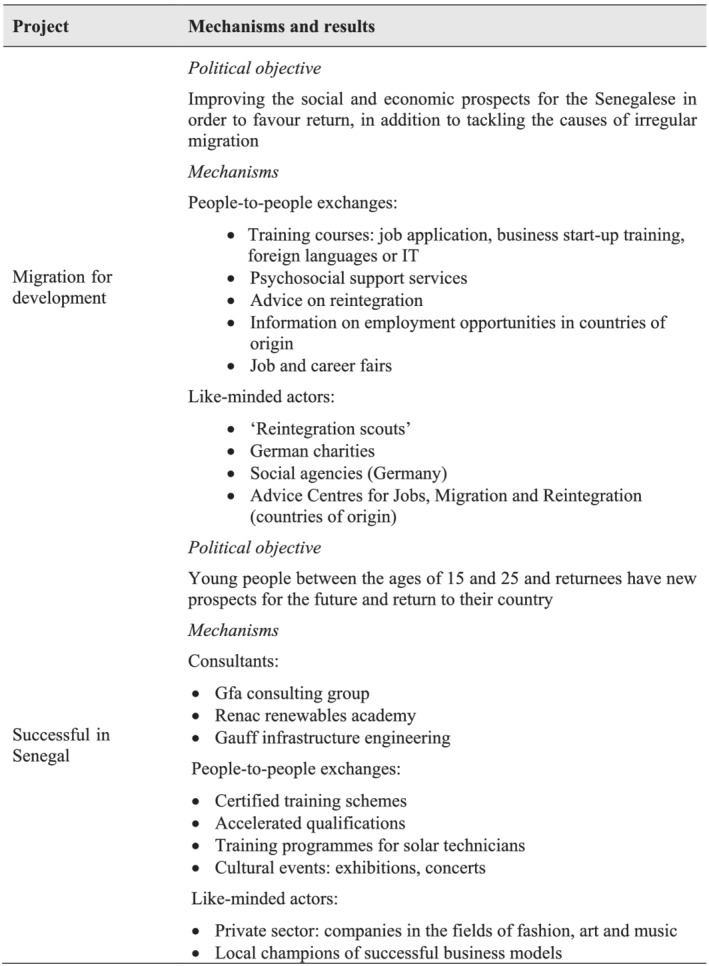
Aid‐influence mechanisms and expected political results in German cooperation migration projects. *Source*: the author

Here, again, the mechanisms at work are people‐to people exchanges and like‐minded actors. Another similarity between this programme and the Spanish cooperation project in Thiaroye is that like‐minded actors are built up from scratch: unlike the DGASE in the first project analysed, both information captains in Senegal and reintegration scouts in Germany are created in the framework of the implementation of the projects (see Figure 5). The fact that different German organisations (such as charities and social agencies) participate in the programme means that it involves some sort of tied aid.

This is also the case with Successful in Senegal, which is linked to the Migration for Development programme. The project's aim is for young Senegalese people aged 15 to 25 and returnees to have new prospects for the future and to return to their home country (GIZ, 2020a). More specifically, it provides training support for job and business opportunities in innovative sectors such as renewable energy, craft trades or the service sector, and has established incubation centres known as ‘Teranga Hubs’. German consultancy firms (gfa, Renac and Gauff) provide training and qualifications via training and technical centres and technical and vocational schools. Cultural events show the potential of creative industries in Senegal (see Figure 5).

As a result, GIZ (2020a) claims, 6 414 young people and returnees have attended training courses for business founders, learning how to calculate budgets and write business plans, and studying the basic principles of successful marketing. Also, 5 238 people have taken advantage of various training and skills enhancement courses on topics such as recycling, the bakery trade and mobile phone repair.

Once again, most of the mechanisms of the aid‐influence nexus identified by academic literature are at work. With the exception of aid conditionality, influence might be conducted, in this particular project, through consultants, other people‐to‐people exchanges and support for like‐minded actors. Moreover, since consulting firms are German, tied aid is also involved. As in the other projects identified for this case study, there are strong intersections between the various mechanisms: consultants interact with like‐minded actors via people‐to‐people exchanges.

As described in the previous section, EU donors in Senegal also aim to disseminate the gender equality agenda. Donors' foreign action is not only driven by interests, such as migration control. Agenda setting, particularly around certain values, has historically been a key part of development cooperation (Engberg‐Pedersen, 2020). Spain claims gender equality to be a core element of its development cooperation. As a result, it has a diverse portfolio of projects that include gender equality goals. The project for the support of the National Observatory of Parity (*Observatoire National de la Parité*, ONP) and the citizen's debates on women participation are part of this aim.

More specifically, according to the project description (ONP, 2018), emulating the international community, Senegal has committed to achieving the Sustainable Development Goals (SDGs) by 2030, including Goal 5 on gender equality and women's empowerment. In other words, influenced by the development ecosystem, this partner country is now committed to the value of gender equity. This is the main goal of the ‘Programme for the support to producers and users of gender statistics in Senegal’, 10 an €840 000 project that aims to support producers and users of gender statistics with a view to contributing to the implementation of the National Strategy for Equity and Gender Equality Awareness. The programme's main objectives are to improve gender sensitivity in Senegal's sectoral and national policy documents and to strengthen the position of the ONP as a reference institution on gender equality information in Senegal (ONP, 2018).

The ONP appears here as a key like‐minded actor. Not only is it embedded in the Senegalese Administration (something that also occurs with the DGASE and with BAOS), but it is also directly under the Presidency of the Republic. The ONP is made up of statistical professionals sharing the strategic view of EU donors as regards gender equality and the importance of disaggregating policy data by gender.

**FIGURE 6:**
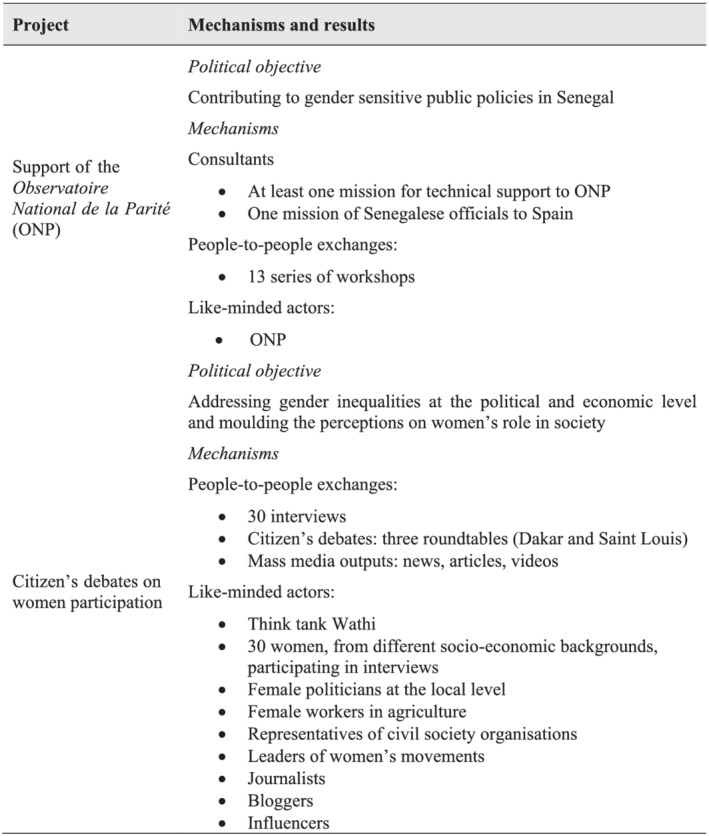
Aid‐influence mechanisms and expected political results in Spain's cooperation projects for gender equity. *Source*: the author

The project, strongly oriented towards capacity building, implies the participation of consultants and people‐to‐people exchanges. As for the latter, at least 13 series of workshops are held in the project's framework, aimed at (a) sharing gender statistic diagnoses and statistical procedure handbooks with technical counterparts, (b) discussing the results of the project with the Administration (Directorate General for the Budget) and the Legislative power (National Assembly) and (c) training technical staff in the production and use of gender‐sensitive statistics. Training activities also include the participation of consultants, with at least two technical missions (see Figure 6).

In a small project worth slightly more than €30 000, Spanish cooperation also supports a series of citizen's debates on women's participation in economic and political life. Through the organisation of three citizens' debates and mass media outputs (interviews, blogs, videos, articles …), the project aims at placing gender inequality at the core of the public debate in Senegal. Consequently, the role of women in economic and political life should be made more visible, and the general perceptions on the role of women in society should thus be moulded (AECID, 2020a).

Unlike the project for supporting the ONP, which aims at building capacities, this action is oriented towards communication outputs. The essence of the project is concerned with amplifying the message not only of like‐minded actors such as the think tank Wathi, the direct beneficiary of the project, but also of female leaders and feminist organisations. This is achieved through different face‐to‐face and digital people‐to‐people exchanges (see Figure 6).

The aim of this study is not to calibrate the extent to which donors influence aid partners. However, once the potential aid‐mechanisms have been identified both theoretically and empirically for these six projects, the obvious question arises as to whether these mechanisms have been (or are being) effective in triggering political dialogue between the parties and leading to some influence exertion on the part of donors, in order to have their objectives, interests and values reached.

This issue was addressed in several interviews with key informants. In general terms, according to the respondents, the existence of aid‐influence mechanisms *per se* does not guarantee a strengthened political dialogue between donors and recipients. In other words, in the framework of this study at least, aid does not systematically lead to influence, despite the existence of particular aid‐influence links. For instance, as was argued by one of the informants, gender equity projects are not having a deep transformative effect on Senegal's political approach to gender issues. Another example in the migration domain is that the National Plan for Migration still has not been officially approved. This happens in spite of having the mechanisms at work and despite walking all the steps (not without difficulties) in the aid projects. As one interviewee put it ‘Senegal signs the international agreements on gender equity, but these are not really implemented’ and ‘there is a superficial implementation of gender equity measures’.

A series of factors were argued for explaining the ineffectiveness of the identified aid‐influence links.

Firstly, the lack of political will of the partner country, which can be due to conflicting interests between donors and partner countries. For instance, while the EU aims at strengthening migration control, Senegal might want the opposite, given the external revenues that come in in the form of remittances. Alternatively, donor and partner country might simply not share the same values, as might happen with differing approaches to gender gaps.

A second reason might be that projects' stakeholders (more specifically like‐minded actors) are not part of the Administration or, if they are, they do not always have an essential role in it. For instance, in the case of the project on promoting education and labour opportunities in Thiaroye, there is a very marginal participation of local authorities, with the key actors being the Senegalese *Association Éducation Solidaire* and the European NGO Open Arms. Something similar happens with the citizen's debates on women's participation, which ends up being more of a civil society reflection rather than a political dialogue between donor and partner where the donor might influence the partner's political vision and agenda. As already described, the ONP is part of the Administration and is also placed at a very high level, linked to the Presidency of Senegal. However, it has more of a monitoring and informative role than an executive and political one, with a mild margin of manoeuvre for actually redirecting gender policies. In that same line, having the DGASE embedded in the Ministry of Foreign Affairs limits its capacity to implement the project's objectives, given the high rotation of the personnel and because, unlike other ministries, the Ministry of Foreign Affairs is not used to dealing with local actors.

A third reason that arose during the interviews is that, in some cases, the people involved in or representing the aid‐influence link are not fit for the projects. For instance, consultants for the project on Migration‐development synergy might not have been well selected, given their insufficient knowledge on the topic.

A fourth reason could be that some potential aid‐influence mechanisms do not involve stakeholders from both donors and partner countries. For instance, in the ONP workshops, all participants are Senegalese.

Lastly, a fifth reason pointed out is the scale of the project. This was mentioned both for the citizen's debates on women's participation (a €30 000 project) and for the project on education and labour opportunities in Thiaroye (at €96 000).

## 5 CONCLUSIONS

This study explores the aid‐influence mechanisms (previously identified by the academic literature) in six aid projects implemented in Senegal by Spain and Germany, with the involvement of the EU, in the two fields of gender equity and migration management. This particular selection shows how the exertion of influence is motivated by a donor's values (which is the case for gender equality) or by a country's interests (in the case of migration management). This leads to the first significant conclusion, which is that the same mechanisms (aid conditionality, tied aid, consultants, people‐to‐people exchanges and the support of like‐minded donors) seem to operate theoretically and empirically, regardless of whether power seeks to export values or interests.

A second conclusion is that, for the particular case of these six projects, some mechanisms (identified in academic literature) prevail over others. Projects include all manner of people‐to‐people exchanges and support for like‐minded donors. However, tied aid and consultants are not that frequent and aid conditionality is non‐existent. This might be explained by the alignment between the donor's political objectives and the projects' goals. If a donor is interested in the broad goal of fostering the gender equality agenda and the specific aid projects seek that same target, there is no need for additional bargaining. This is a rather different situation to that of Japan or South Korea exchanging votes in multilateral spaces for access to fisheries, for instance. An alternative explanation could be that political conditionality does not manifest at the project level but rather at a higher political level. The mere existence of bilateral aid programmes might involve some type of political conditionality on the part of donors.

Thirdly, the presence of aid‐influence mechanisms does not lead, inextricably, to power exertion on the part of the donor country over the aid partner. A series of factors might ‘short‐circuit’ the link, such as the lack of political will on the part of the partner, the insufficient involvement of its Administration or local actors, the mis‐selection of people involved in the aid‐influence link, or the scale of the project. This result could be considered a relevant input for the academic debate on the exertion of international power through aid, since such influence has been sometimes taken for granted or considered automatic in a particular system.

A final observation is that the research for this paper shows varied forms of support to like‐minded donors. Different types of partners are targeted by donor countries: pre‐existing private initiatives (feminist associations, for instance), the public administration or even like‐minded actors built from scratch and as a result of these projects or of pre‐existing aid projects (captains of information or reintegration scouts). Future research could explore the extent to which support to different types of like‐minded actors involves more or less effective aid‐influence links. Supporting public bodies (ONP, DGASE and BAOS) means donor countries ‘infiltrating’ the partner country's political domain (to put it in Anderson's, 2018, terms). In a similar vein, the creation of stakeholders for the purpose of the project or programme implies the possibility for the donor to mould such partners. It could be argued that these two modalities might provide greater leverage for the donor to influence the partner country than when supporting private initiatives.

## Data Availability

Not available.

## Footnotes

^1^
See, for instance, Carmody (2012), Melber (2013), Villegas Mendoza (2015), Hwang (2014), Deych (2015), Gosovic (2016), Nayyar (2016), Kevany (2014) or Beech et al. (2015).

^2^
Gender equality is here used when referring to the objective to be achieved (e.g., socio‐economic conditions of women improved), while gender equity refers to the actions or means for attaining this goal (e.g., development cooperation initiatives for improving the socio‐economic conditions of women).

^3^
Being the latter the main tool identified by Harmer et al. (2013) for the aid domain.

^4^
Other paradigms or agendas strongly linked to international assistance are those of gender equality (Engberg‐Pedersen, 2020; Fejerskov, 2017), social policy (Kwon et al., 2015) or global health (Dauvergne & Farias, 2012).

^5^
It is worth noting that the influential capacity of the EU in the aid domain has also been studied at the European level: how MS shape their aid according to the European doctrine (Carbone & Orbie, 2019; Olivié & Pérez, 2020).

^6^
This input by Sandor (2016) shows a certain convergence of the Foucauldian approach to disciplinary power and the exertion of power seen from the Realist tradition in this particular domain of international exchanges.

^7^
Author's translation.

^8^
Author's translation.

^9^
Author's translation.

^10^
Author's translation.
